# Supplementary material for: Thermodynamic Profiling Reveals DNA Polymerase Template Binding, Substrate Incorporation, and Exonuclease Function
Source: Int J Mol Sci. 2025 Dec 10;26(24):11909. doi: 10.3390/ijms262411909 (PMC12732654; doi:10.3390/ijms262411909)
Supplement: Supplementary file 1 [file ijms-26-11909-s001.zip › supplementary Table 1,2.pdf]

Supplementary Table 1: ITC data of SS_01 binding to primed templates in the absence or presence of catalytic metal ions.

	KD (nM)	ΔH (kcal/moL)	ΔG (kcal/moL)	-TΔS (kcal/moL)
without Mg^{2+}	129 ± 9.46	-22.37 ± 4.56	-9.41 ± 0.06	12.93 ± 4.59
with Mg^{2+}	243 ± 80.53	-28.37 ± 7.01	-9.01 ± 0.23	19.36 ± 7.17

Supplementary Table 2: ITC data for the binding affinity of SS_01 and primed templates in the presence of Mg^{2+} or Ca^{2+}.

	KD (nM)	ΔH (kcal/moL)	ΔG (kcal/moL)	-TΔS (kcal/moL)
with Mg^{2+}	239 ± 68.23	-74.47 ± 7.27	-9.04 ± 0.184	65.37 ± 7.41
with Ca^{2+}	317 ± 75.23	-46.17 ± 5.33	-8.85 ± 0.14	37.20 ± 5.80
