# Supplementary figures and images for: Thermodynamic Profiling Reveals DNA Polymerase Template Binding, Substrate Incorporation, and Exonuclease Function
Source: Int J Mol Sci. 2025 Dec 10;26(24):11909. doi: 10.3390/ijms262411909 (PMC12732654; doi:10.3390/ijms262411909)

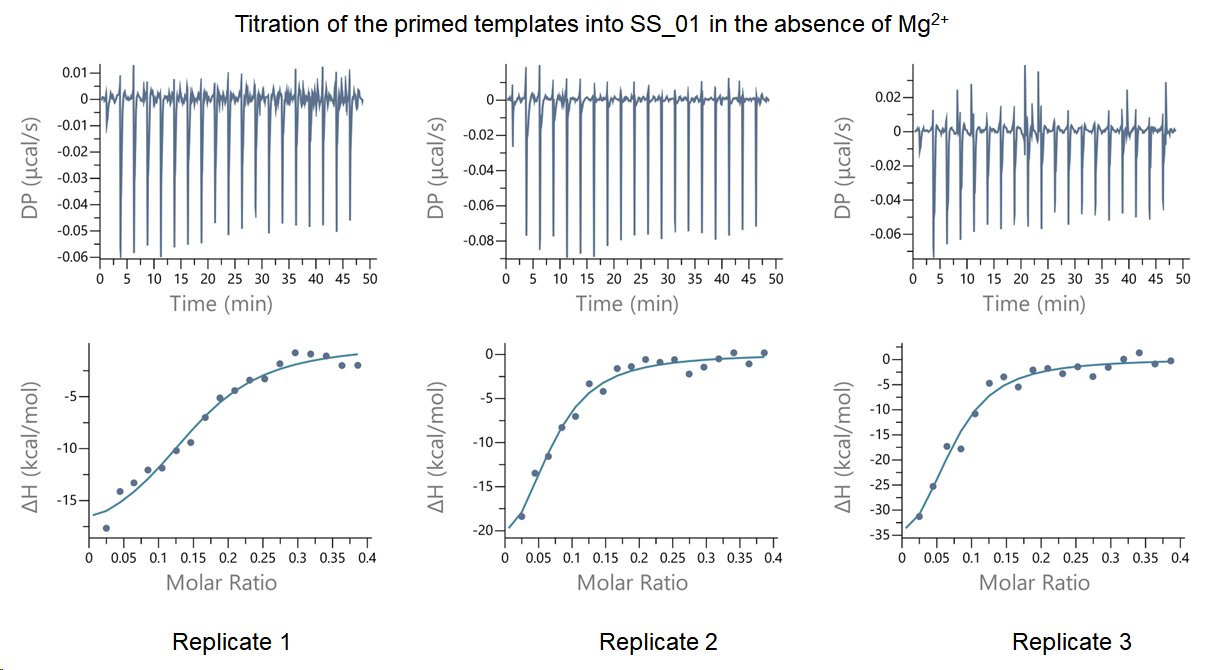

Supplement: Supplementary file 1 [file ijms-26-11909-s001.zip › Supplementary Figure 1.tif]

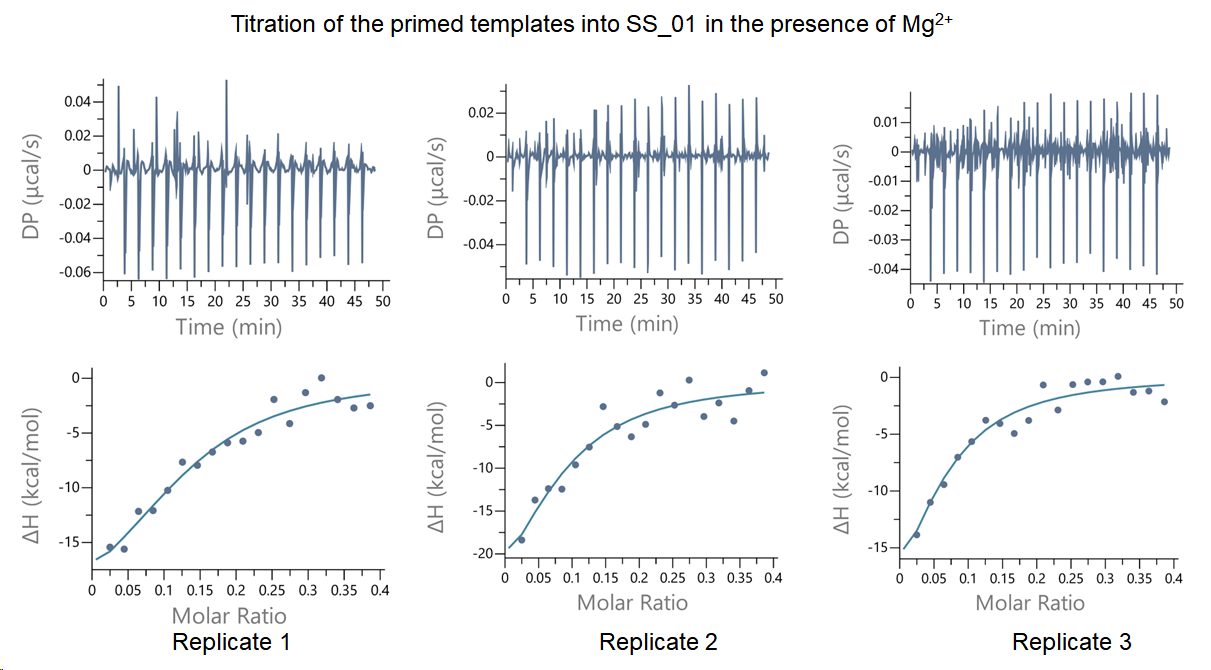

Supplement: Supplementary file 1 [file ijms-26-11909-s001.zip › Supplementary Figure 2.tif]

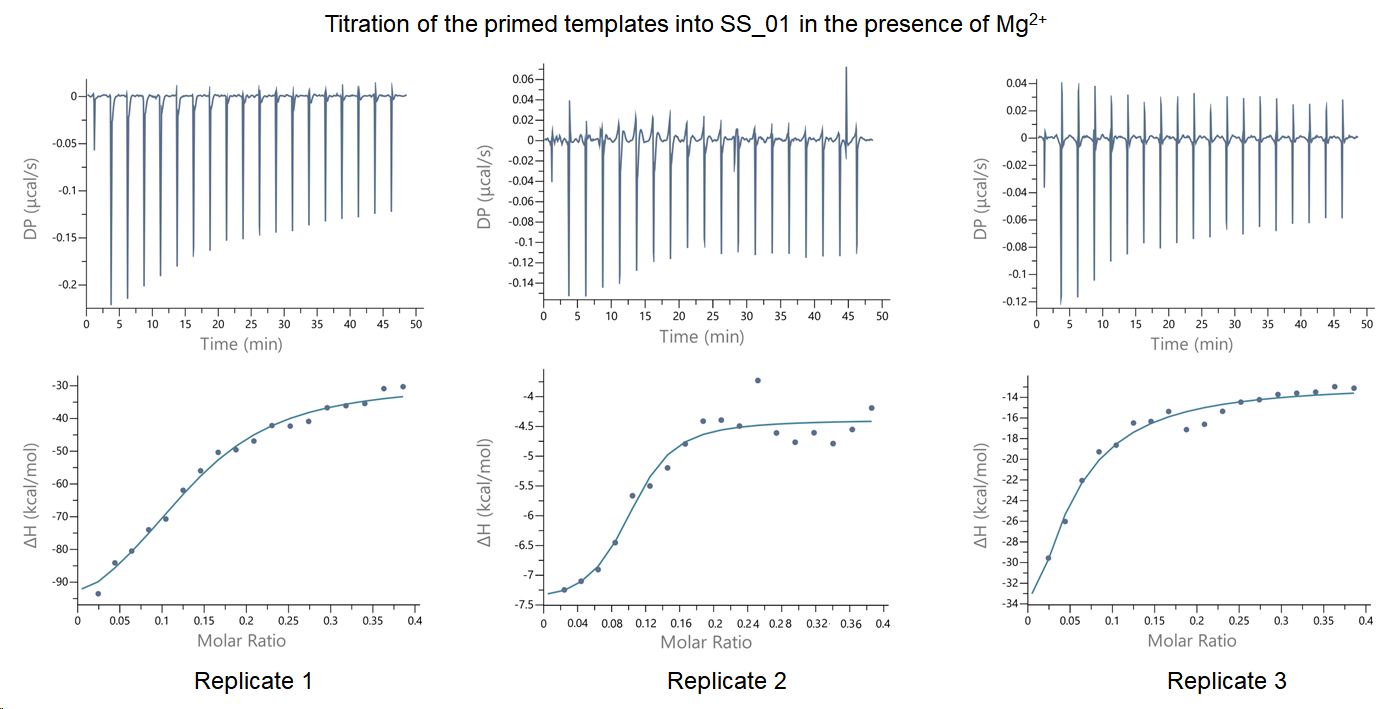

Supplement: Supplementary file 1 [file ijms-26-11909-s001.zip › Supplementary Figure 3.tif]

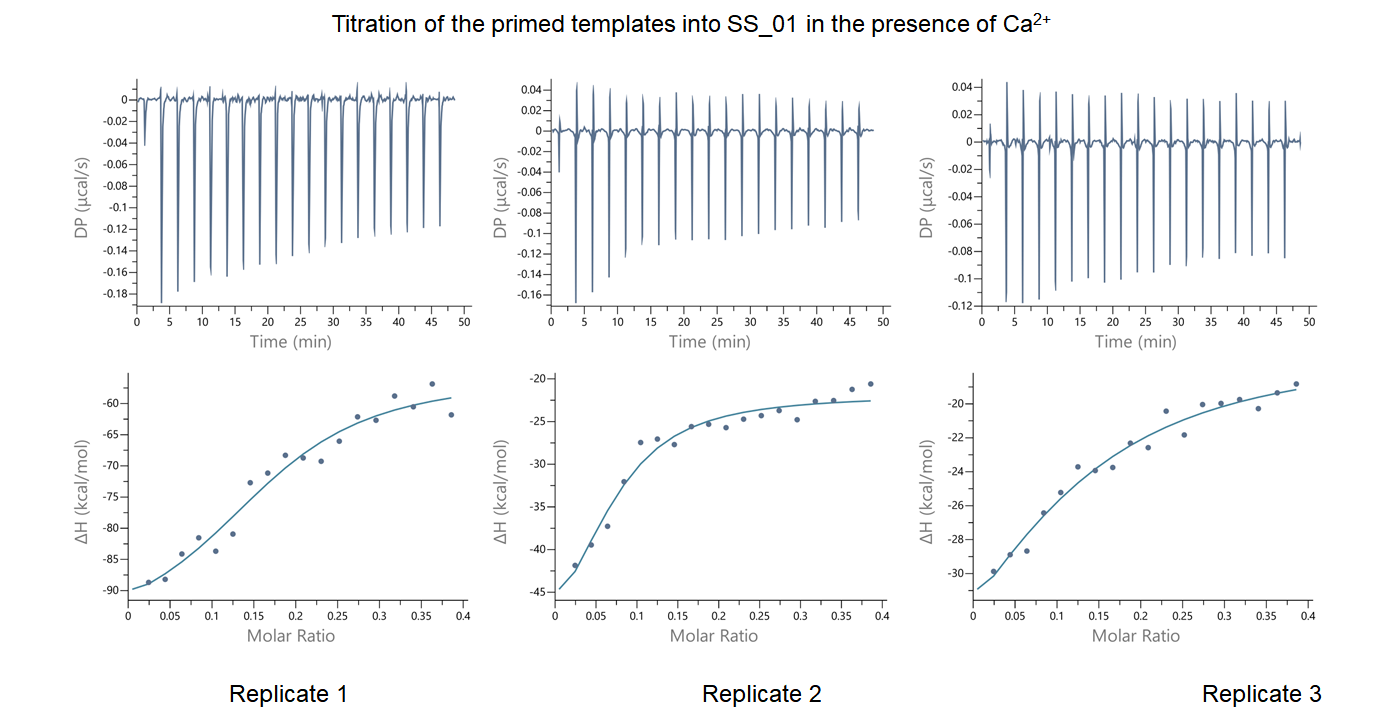

Supplement: Supplementary file 1 [file ijms-26-11909-s001.zip › Supplementary Figure 4.tif]

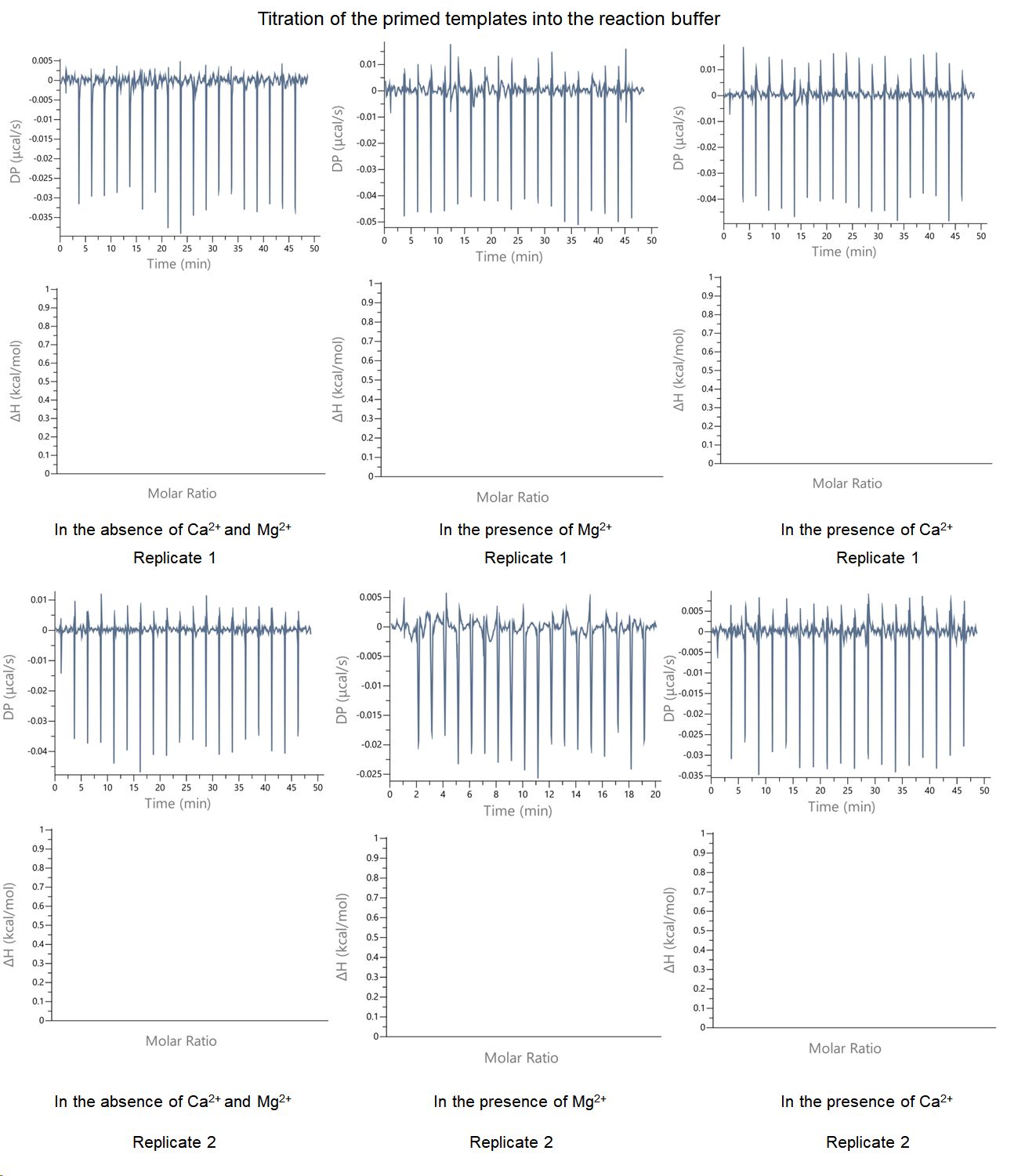

Supplement: Supplementary file 1 [file ijms-26-11909-s001.zip › Supplementary Figure 5.tif]

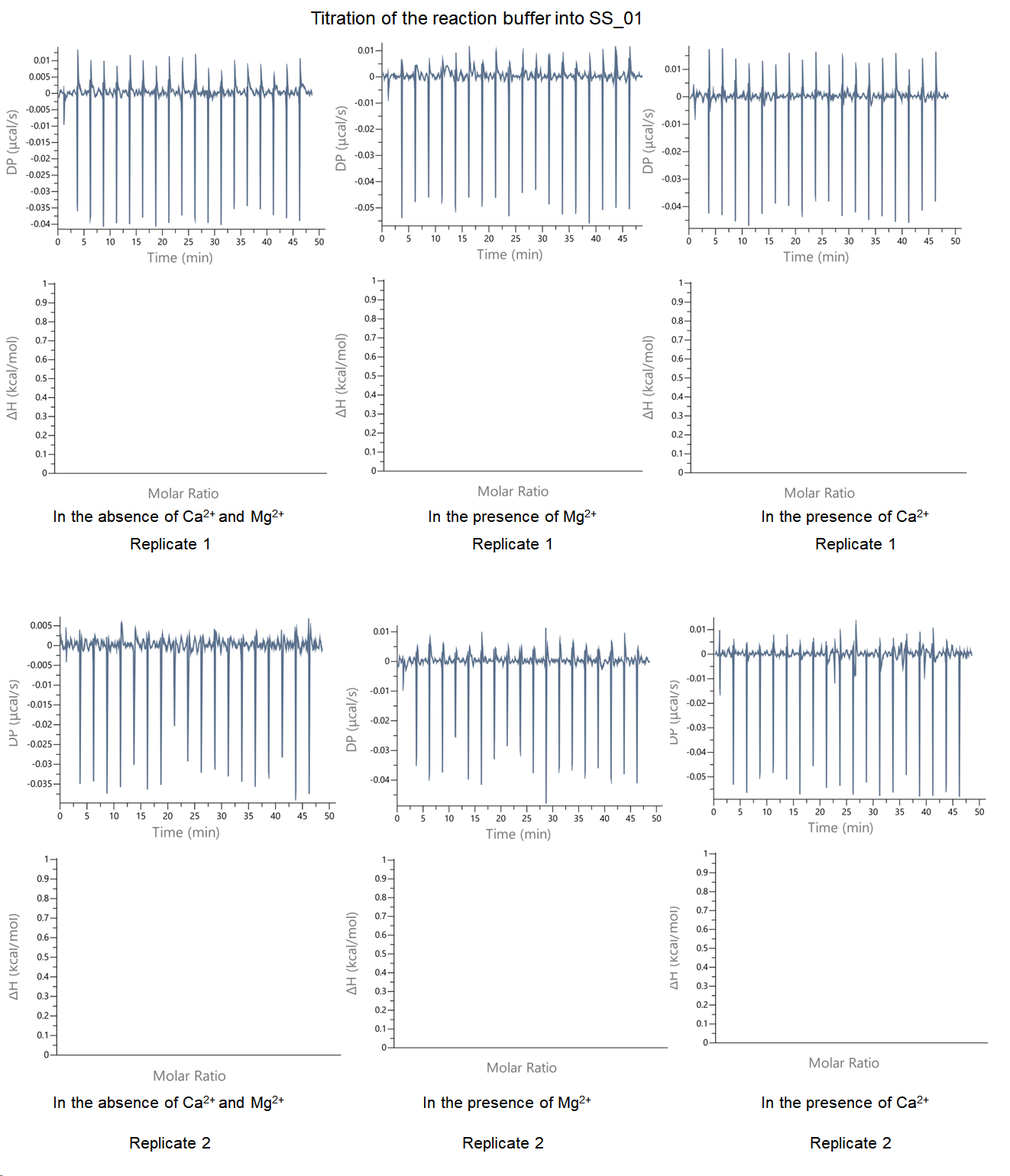

Supplement: Supplementary file 1 [file ijms-26-11909-s001.zip › Supplementary Figure 6.tif]

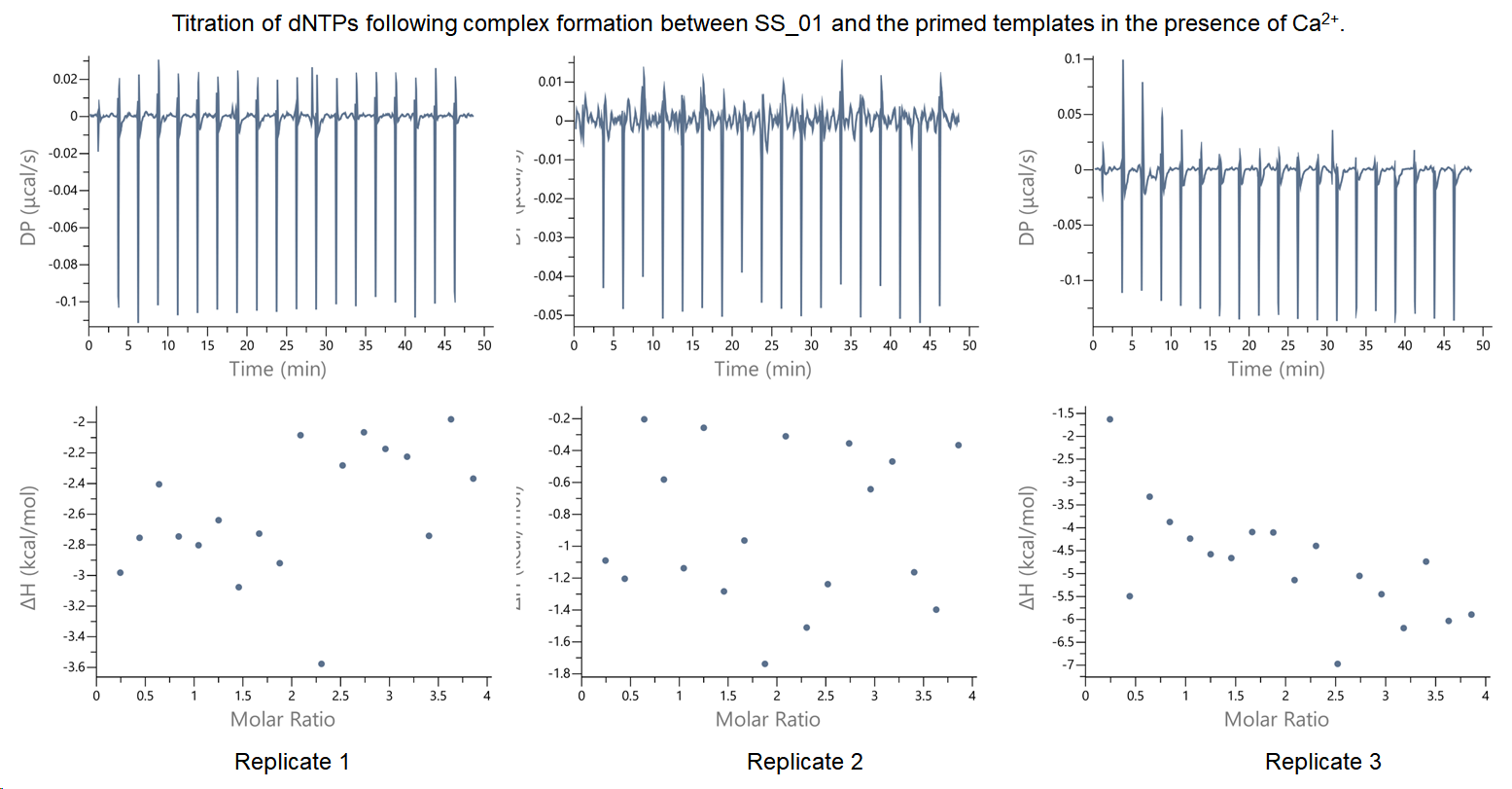

Supplement: Supplementary file 1 [file ijms-26-11909-s001.zip › Supplementary Figure 7.tif]

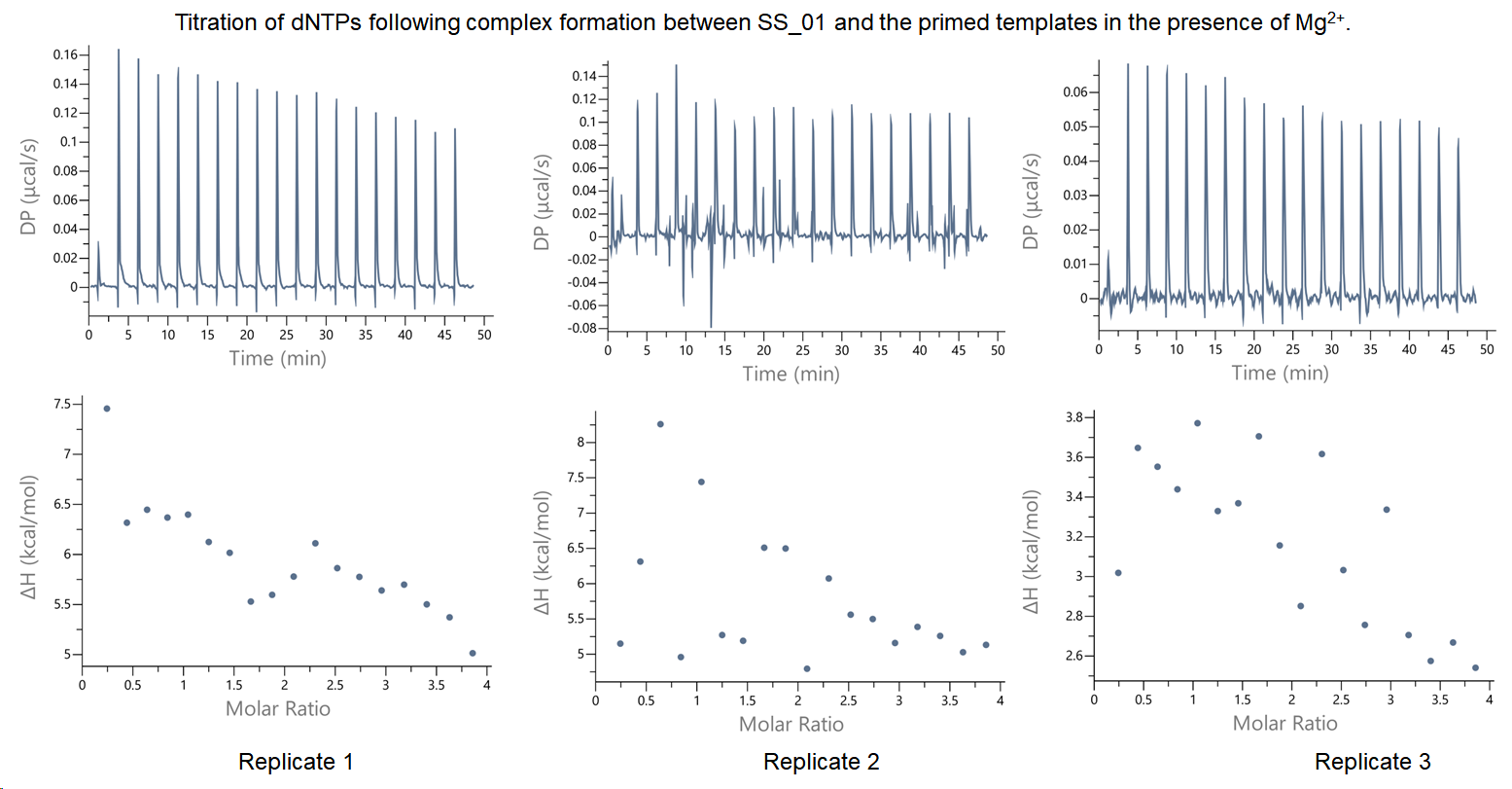

Supplement: Supplementary file 1 [file ijms-26-11909-s001.zip › Supplementary Figure 8.tif]

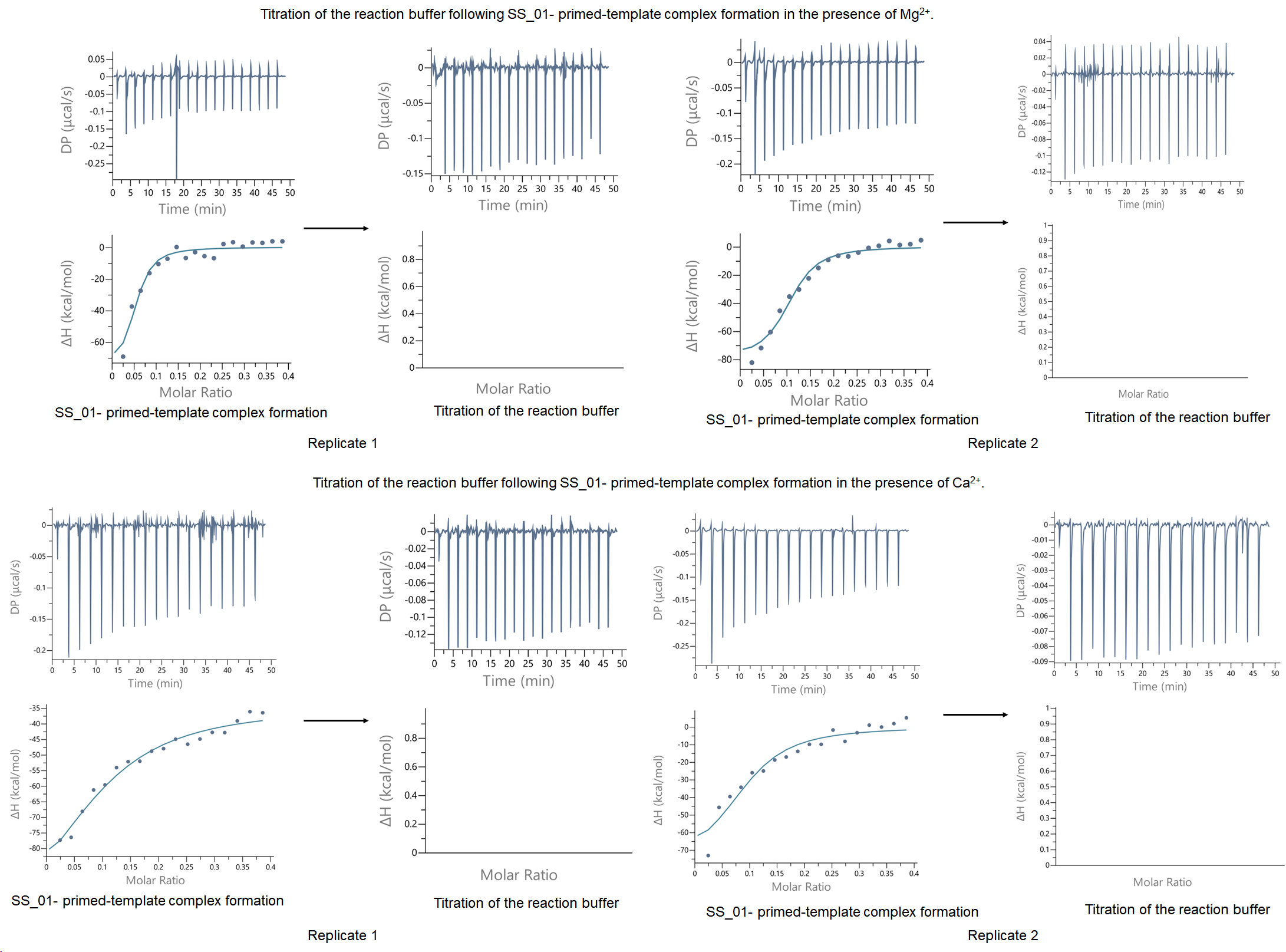

Supplement: Supplementary file 1 [file ijms-26-11909-s001.zip › Supplementary Figure 9.tif]

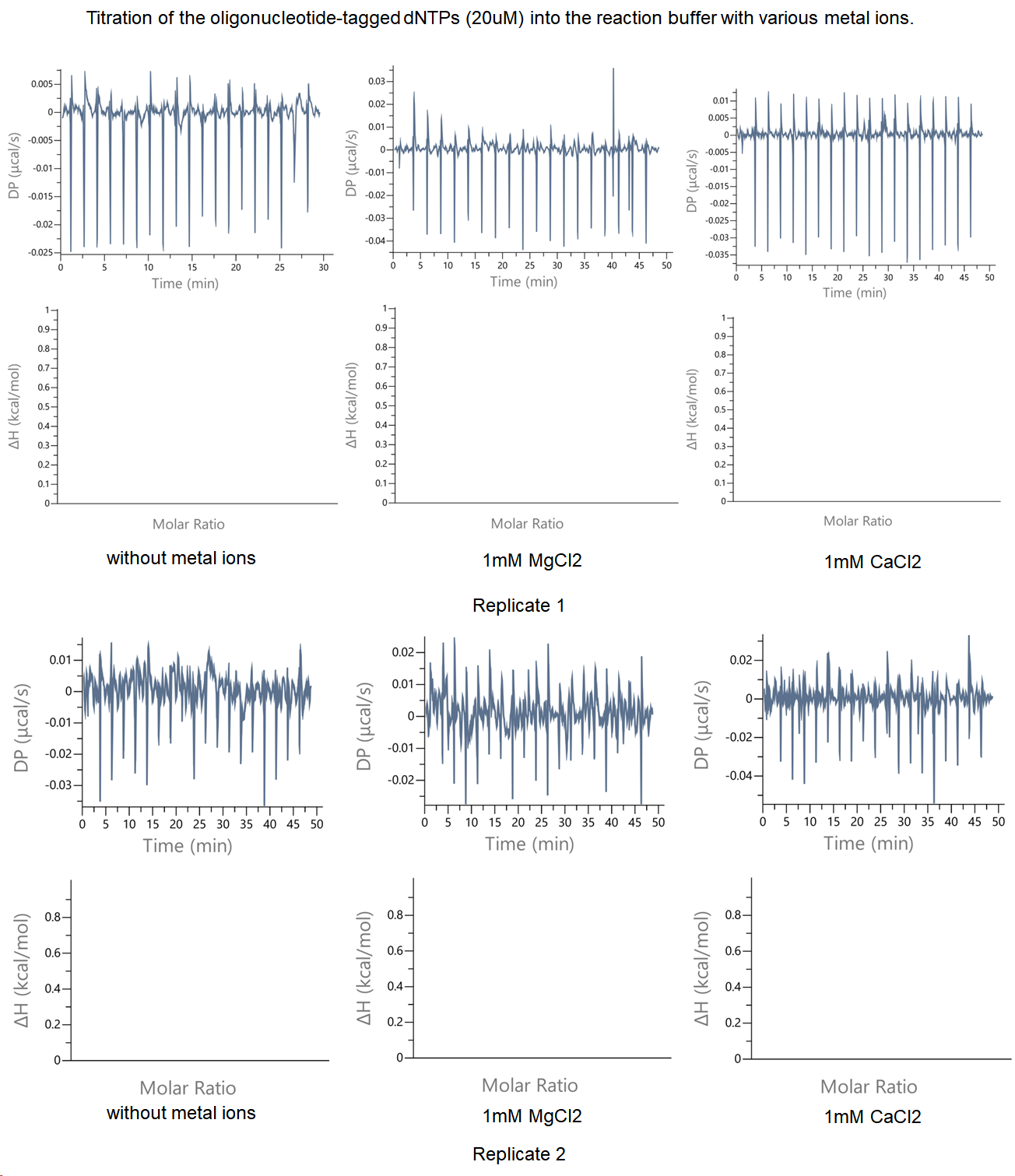

Supplement: Supplementary file 1 [file ijms-26-11909-s001.zip › Supplementary Figure 10.tif]

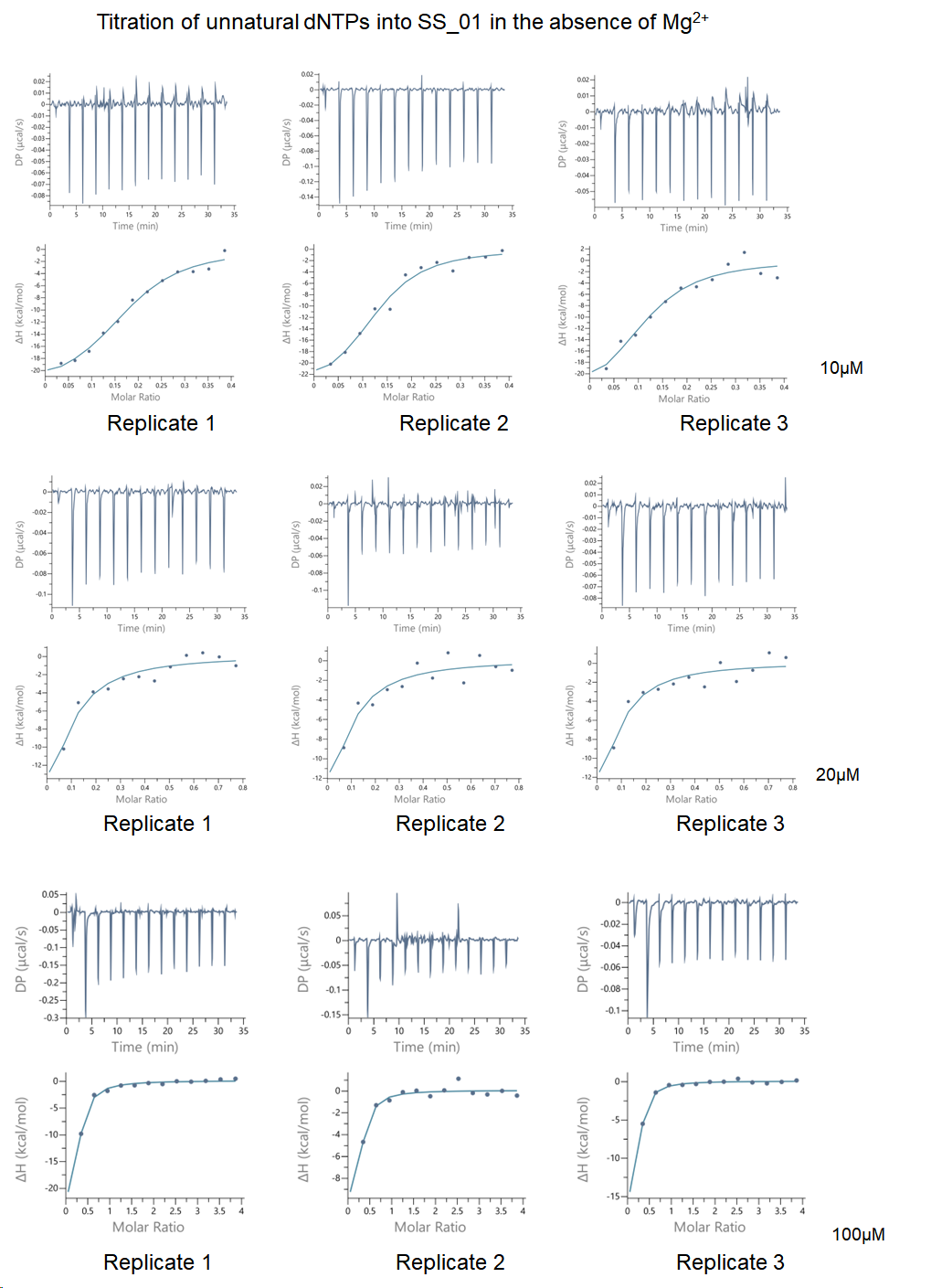

Supplement: Supplementary file 1 [file ijms-26-11909-s001.zip › Supplementary Figure 11.tif]

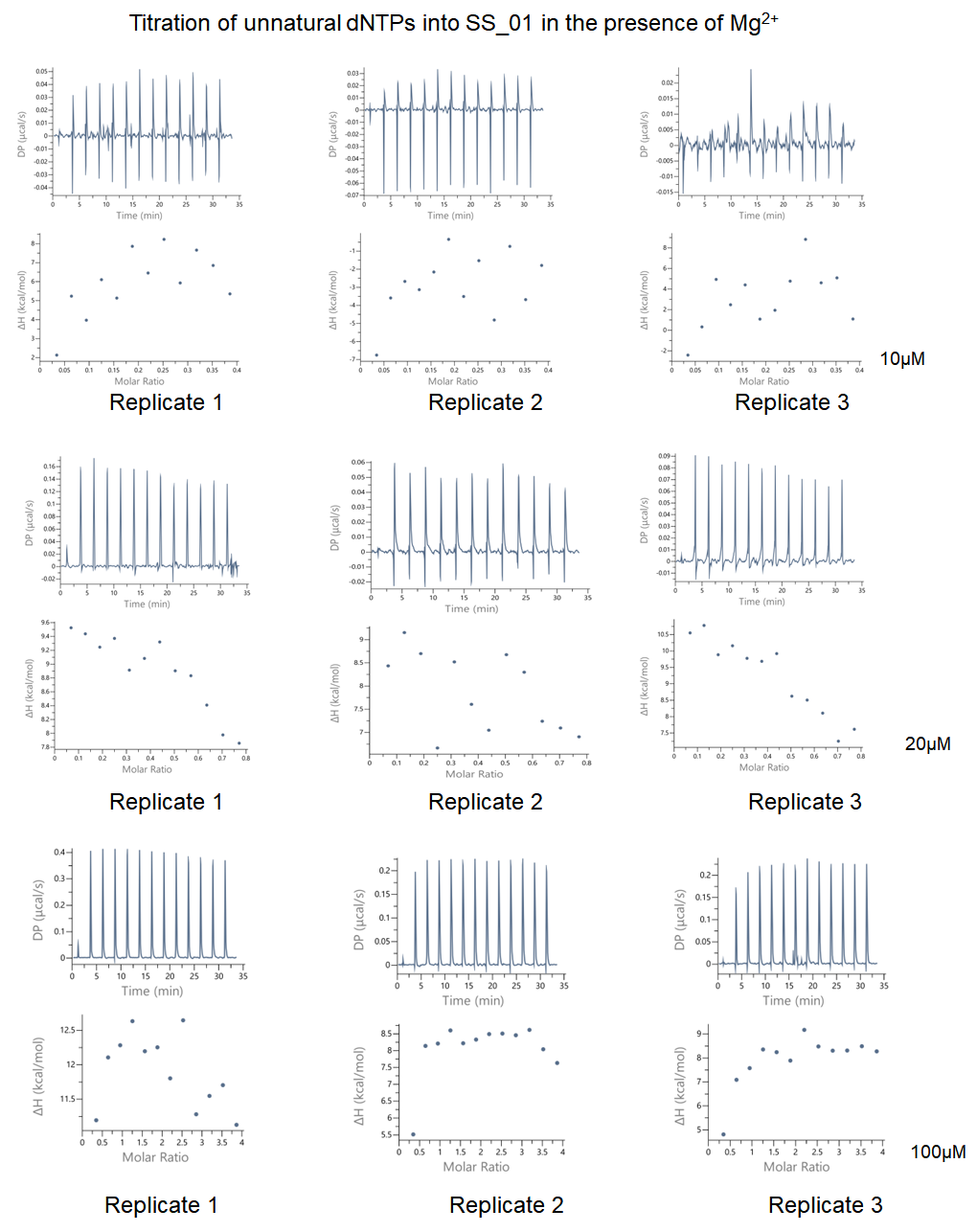

Supplement: Supplementary file 1 [file ijms-26-11909-s001.zip › Supplementary Figure 12.tif]

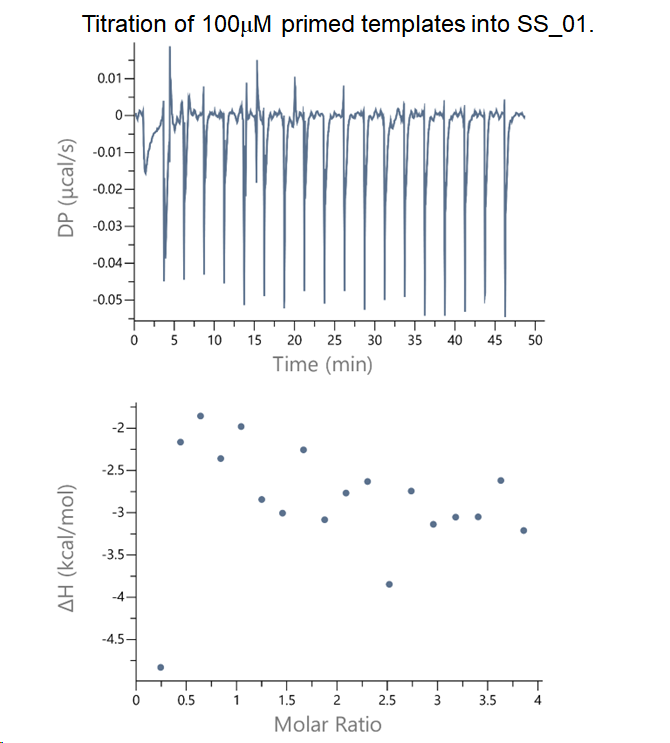

Supplement: Supplementary file 1 [file ijms-26-11909-s001.zip › Supplementary Figure 13.tif]

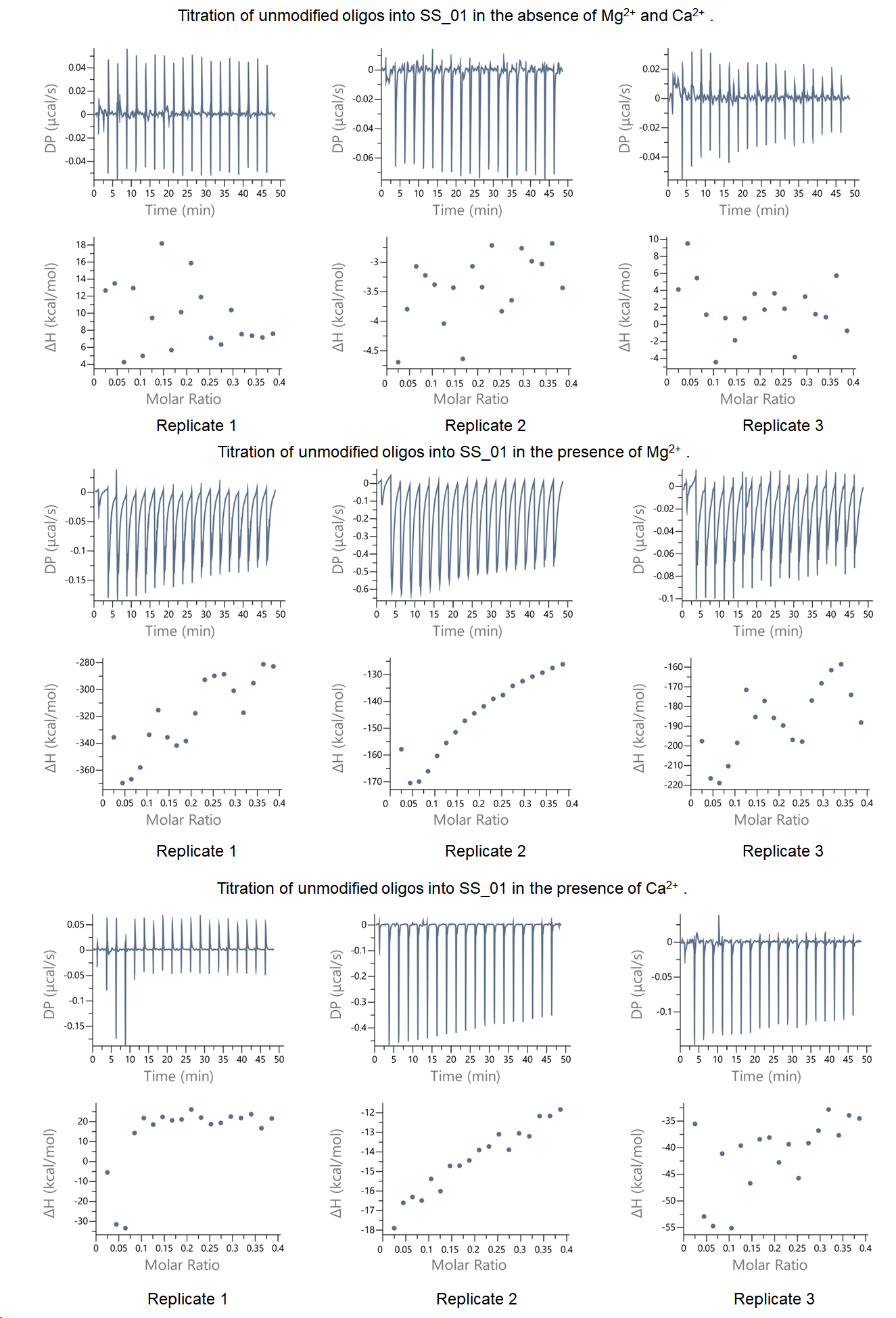

Supplement: Supplementary file 1 [file ijms-26-11909-s001.zip › Supplementary Figure 14.tif]

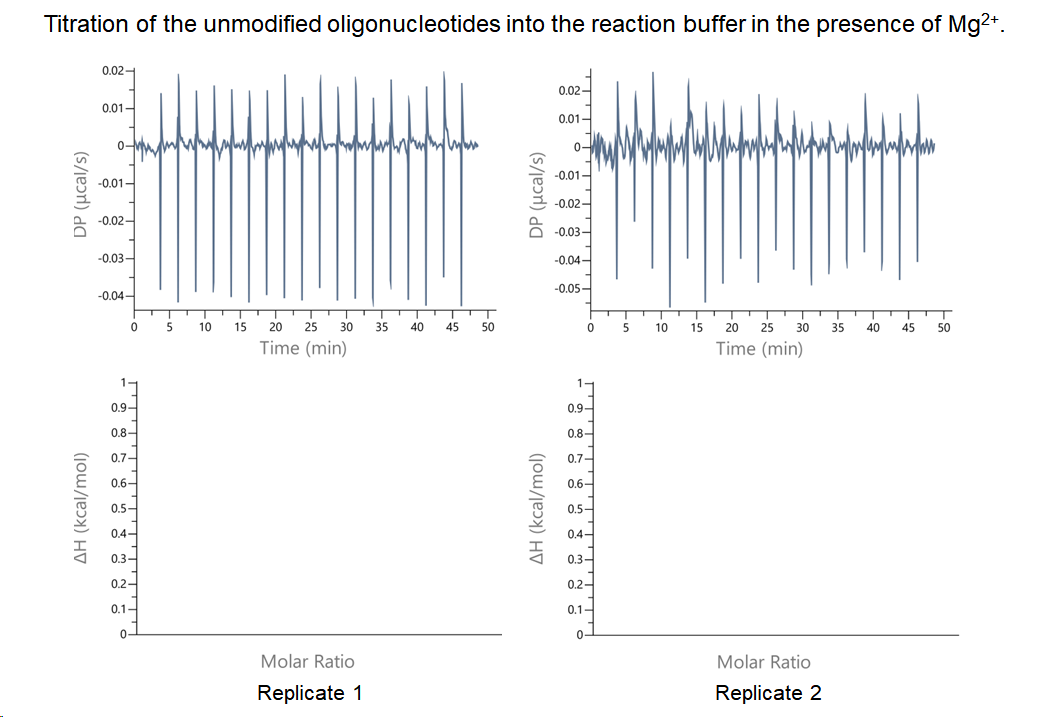

Supplement: Supplementary file 1 [file ijms-26-11909-s001.zip › Supplementary Figure 15.tif]

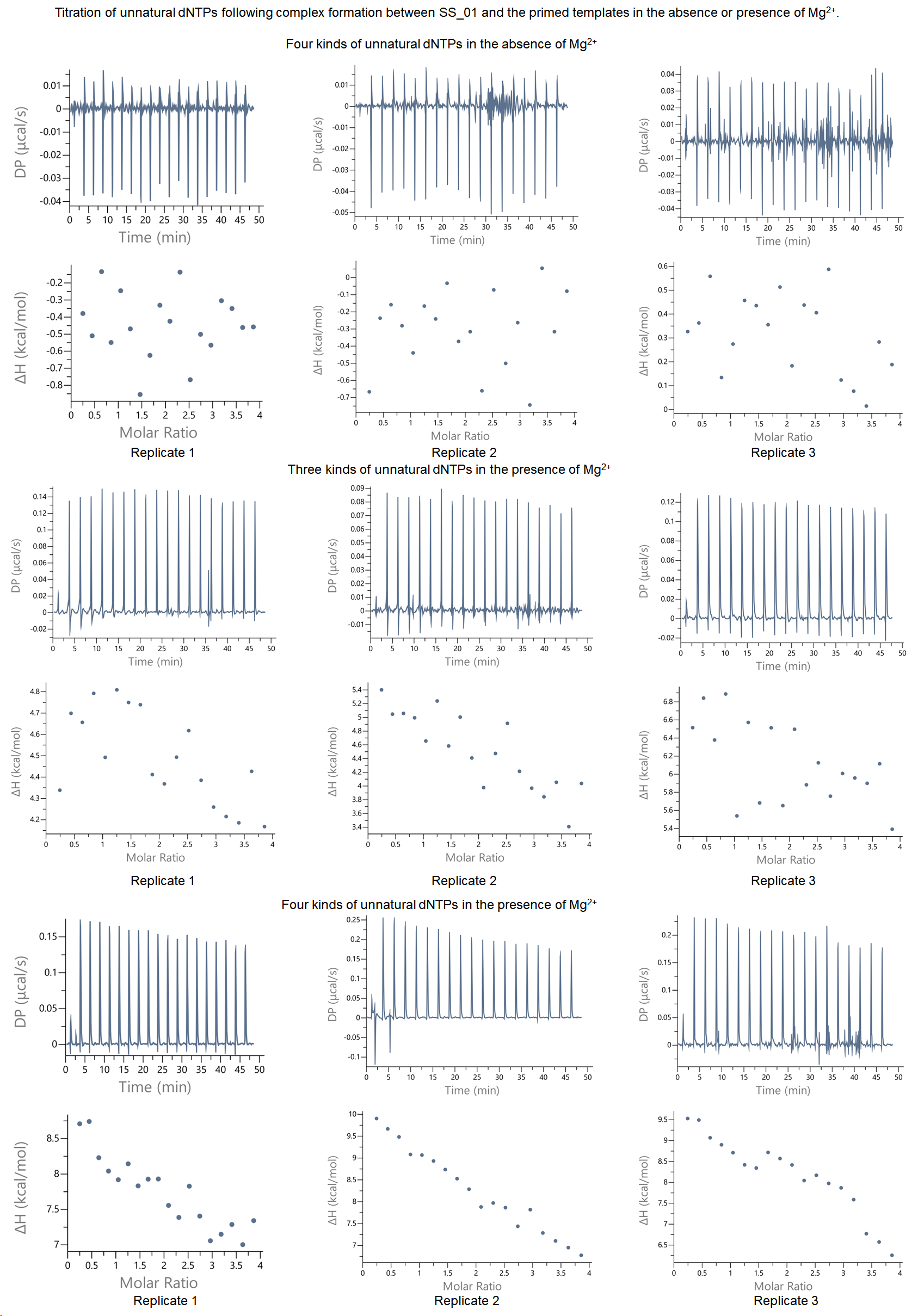

Supplement: Supplementary file 1 [file ijms-26-11909-s001.zip › Supplementary Figure 16.tif]

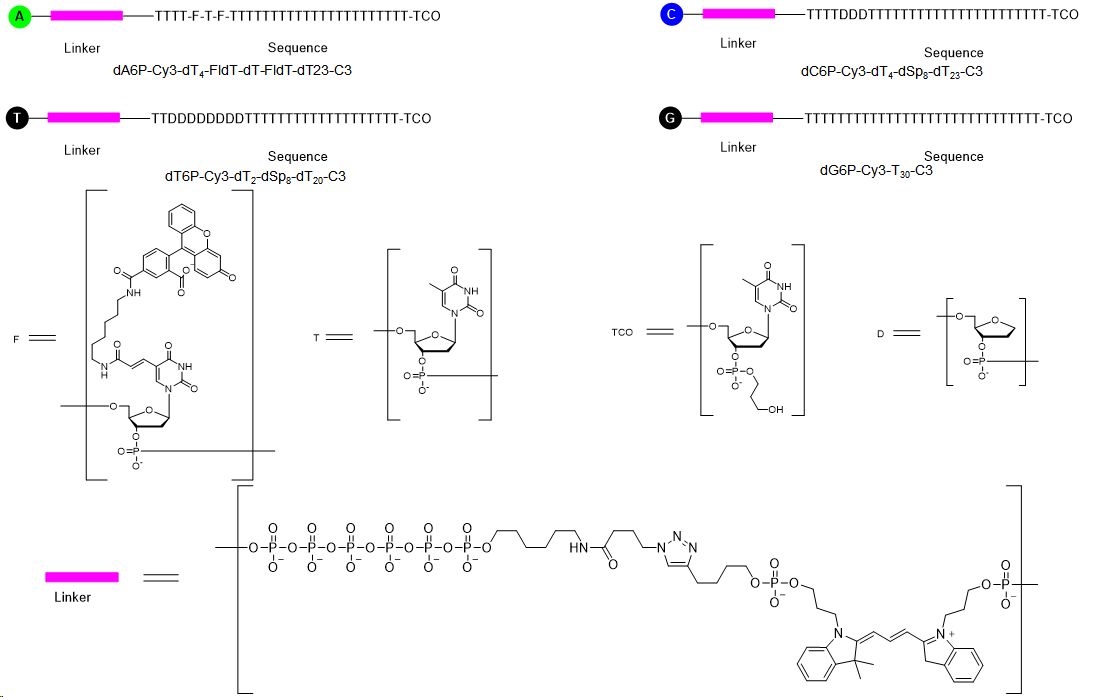

Supplement: Supplementary file 1 [file ijms-26-11909-s001.zip › Supplementary Figure 17.tif]

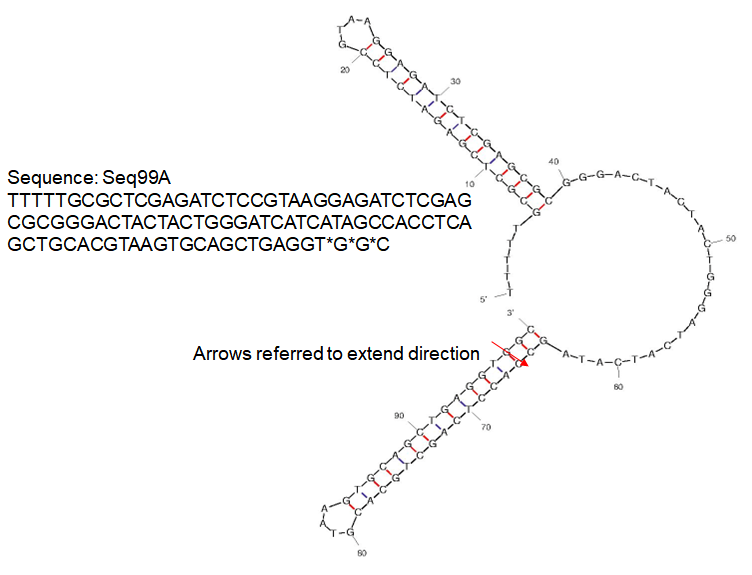

Supplement: Supplementary file 1 [file ijms-26-11909-s001.zip › Supplementary Figure 18.tif]
